# Effect of alcohol consumption on breast cancer: probabilistic bias analysis for adjustment of exposure misclassification bias and confounders

**DOI:** 10.1186/s12874-023-01978-6

**Published:** 2023-07-04

**Authors:** Reza Pakzad, Saharnaz Nedjat, Hamid Salehiniya, Nasrin Mansournia, Mahyar Etminan, Maryam Nazemipour, Iraj Pakzad, Mohammad Ali Mansournia

**Affiliations:** 1 Department of Epidemiology, Faculty of Health, Ilam University of Medical Sciences, Ilam, Iran; 2 Student Research Committee, Ilam University of Medical Sciences, Ilam, Iran; 3 Department of Epidemiology and Biostatistics, School of Public Health, Tehran University of Medical Sciences, PO Box: 14155-6446, Tehran, Iran; 4 Department of Epidemiology and Biostatistics, School of Health, Birjand University of Medical Sciences, South Khorasan, Iran; 5 Department of Endocrinology, School of Medicine, AJA University of Medical Sciences, Tehran, Iran; 6 Departments of Ophthalmology and Visual Sciences, Medicine and Pharmacology, University of British Columbia, Vancouver, Canada; 7 Department of Microbiology, School of Medicine, Ilam University of Medical Sciences, Ilam, Iran

**Keywords:** Probabilistic bias analysis, Alcohol consumption, Breast cancer, Monte-Carlo sensitivity analysis, Population attributable fraction

## Abstract

**Purpose:**

This study was conducted to evaluate the effect of alcohol consumption on breast cancer, adjusting for alcohol consumption misclassification bias and confounders.

**Methods:**

This was a case-control study of 932 women with breast cancer and 1000 healthy control. Using probabilistic bias analysis method, the association between alcohol consumption and breast cancer was adjusted for the misclassification bias of alcohol consumption as well as a minimally sufficient set of adjustment of confounders derived from a causal directed acyclic graph. Population attributable fraction was estimated using the Miettinen’s Formula.

**Results:**

Based on the conventional logistic regression model, the odds ratio estimate between alcohol consumption and breast cancer was 1.05 (95% CI: 0.57, 1.91). However, the adjusted estimates of odds ratio based on the probabilistic bias analysis ranged from 1.82 to 2.29 for non-differential and from 1.93 to 5.67 for differential misclassification. Population attributable fraction ranged from 1.51 to 2.57% using non-differential bias analysis and 1.54–3.56% based on differential bias analysis.

**Conclusion:**

A marked measurement error was in self-reported alcohol consumption so after correcting misclassification bias, no evidence against independence between alcohol consumption and breast cancer changed to a substantial positive association.

**Supplementary Information:**

The online version contains supplementary material available at 10.1186/s12874-023-01978-6.

## Introduction

Breast cancer is the leading cause of death from cancer in women across the world accounting for 25% of the total new cancer cases and 15% of the total deaths from cancer [1]. The incidence of breast cancer varies up to five time in different parts of the world, being higher in developed countries; however, its incidence is on the rise in less developed countries, too [2]. Several studies investigated the risk factors of breast cancer and found that factors like childbearing, advanced age, high menopause age, low menarche age, low physical activity, high-fat diets, high BMI, positive family history, nulliparity, use of OCP, and smoking could play a role in its occurrence [2–6].

Alcohol consumption, as one of the risk factors of breast cancer, has drawn researchers’ attention in the past decade. However, there is still controversy about the association between alcohol consumption and breast cancer; some primary studies found a positive relationship [7–10] while others rejected any association [11–16]. Several researchers conducted different meta-analysis studies to address this controversy. Although most of these meta-analyses indicated a positive association [17–24], the majority were very weak [19, 20, 22–25]. This is while some other studies rejected any association between moderate alcohol consumption and breast cancer [25]. On the other hand, in addition to the fact that most of the studies failed to adjust for important confounders [24, 25], they also suffer from alcohol consumption misclassification due to self-reporting [17, 22, 23].

It is clear that the alcohol consumption may be underreported due to its social stigma. Several studies have found misclassification in alcohol consumption reporting [17, 22, 23], which may lead to biased effect estimates [26, 27] of alcohol consumption on breast cancer, explaining the contradictory results mentioned above. Therefore, statistical methods have been suggested to be used to correct misclassification bias secondary to self-reported alcohol consumption [28].

In general, two approaches have been developed to correct misclassification: Probabilistic Bias Analysis Method (PBAM) by Lash and Fox [29, 30], and Bayesian Method [31], by MacLehose [32] and Gustafson [11]. Both models can control the measurement bias but the PBAM, which is based on the Monte-Carlo simulation [12, 29, 30], is conceptually simpler and easier to perform. Studies have shown that in the case of selecting similar priors, the results of both models may be similar [33].

Simple bias analysis and multidimensional analysis [34] perform bias correction by using a set of few bias parameter (sensitivity and specificity) values, while PBAM creates simulation intervals that are adjusted for a probability distribution of bias parameters as well as random error and confounders through record-level correction of the misclassified exposure [30]. The general PBAM approach of Fox et al. [29] and Lash et al. [30] was developed for polytomous exposure variables.

Although several studies investigated the association between alcohol consumption and breast cancer [7–25], none of them have adjusted for the measurement bias secondary to the self-reported alcohol consumption. Therefore, this study was done to assess the effect of alcohol consumption on breast cancer after correcting alcohol consumption misclassification bias and adjusting for a set of confounders using PBAM.

## Materials and methods

### Design and sampling

This case-control study was performed in Tehran, Iran. The methodological details of the present study have already been published previously [35]. This study recruited 1000 patients with breast cancer as case, selected in an ongoing manner (incidence cases) from breast cancer detection clinics in Tehran, Iran, whose disease was diagnosed and confirmed by pathological study and/or a specialist and the same number of individuals without cancer as control, selected from the general population of all Tehran districts through proportional-to-size stratified random sampling. Cases included breast cancer patients aged 25–75 years old that expressed willingness to participate in the study and lived in Tehran. The exclusion criteria were pregnancy, other cancers in addition to breast cancer, and healthy women receiving preventive treatments for breast cancer. The study objectives were explained to the subjects and signed informed consent was obtained from all. The data collection tool was a researcher-made questionnaire with confirmed validity and reliability. However, we note that the misclassification problem in the question of alcohol consumption, which is closely related to the construct validity, exists as the question was subject to recall and under-reporting biases. A trained female research assistant made clinical measurements including weight and height. The questionnaire had seven sections, including (1) demographic and general data, (2) physical activity, (3) cigarettes and tobacco use as well as alcohol consumption, (4) diet, (5) pregnancy and past medical history data (history of breast diseases as well as the history of pregnancy along with the delivery date), (6) family history, and (7) clinical measurements including weight and height, weight at puberty (age 12), and weight at 20 and 30 years of age.

### Statistical analysis

**Fig. 1:**
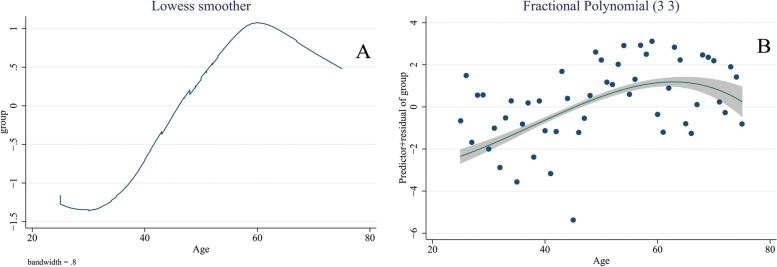
LOWESS (**A**) and fractional polynomial plot (**B**) for the association between age and breast cancer

Some packages in the R software including foreign, doParallel, foreach, triangle, readstata13, MASS, Haven and SUMMER were used for statistical analysis. The relevant literature was searched to prepare a list of confounders. The DAGitty package was used to generate a casual directed acyclic graph (cDAG) [36–46]. The Pearl’s back-door criterion was applied to identify a minimally sufficient set for confounding adjustment [47]. Then, a conventional multivariable logistic regression model was fitted to assess the association of alcohol consumption and breast cancer, adjusted for the set of confounders and the result was reported as adjusted OR with 95% confidence interval [48, 49]. Locally weighted scatterplot smoother (LOWESS) and fractional polynomials were used to determine the appropriate scale for age [50]. Figure 1 presents the LOWESS and fractional polynomial plot for the association between age and breast cancer.

### Bias analysis using PBAM

**Fig. 2:**
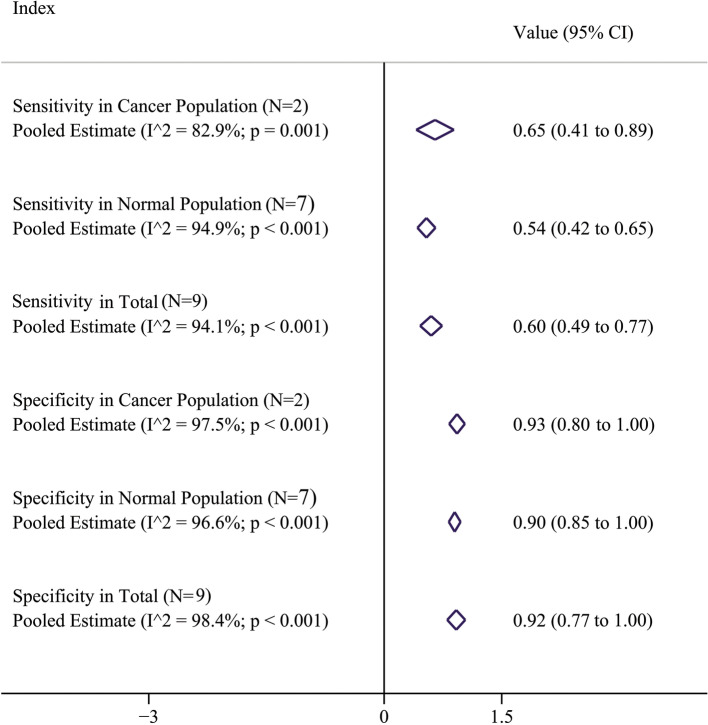
Pooled estimates of specificity and sensitivity; pooled estimates in total was used for non-differential bias analysis and pooled estimates in cancer and normal subjects was used for differential bias analysis

- Step 1: A systematic literature review (without time and language restriction) was done in Scopus, PubMed, and Web of Science to determine the sensitivity and specificity of the question asking about self-reported alcohol consumption, using the following keywords: “sensitivity”, “specificity”, “self-reported alcohol consumption”, “validity”, “accuracy”, “measurement error” and “measurement bias”. The retrieved studies were screened in three stages, including titles, abstracts, and full texts. All the articles that reached the final stage were read carefully and the information such as sensitivity and specificity along with their confidence intervals, gold standard method, were collected. Then, an inverse-variance weighted random-effects model was applied to merge the results [51].
- Step 2: According to the results of the systematic review, nine studies (Supplement 1) were included in the final analysis [52–60] two of which were done in cancer patients [52, 53] and seven were performed in the normal population [54–60]. The pooled estimate of specificity (95% CI) in cancer patients, normal population, and total were 93% (80, 100), 90% (85, 100), and 92% (77, 100), respectively. The estimates for sensitivity (95% CI) were 65% (41, 89), 54% (42, 65) and 60% (49, 77); respectively (Fig. 2).

- Step 3: The probability distributions (including Triangular, Beta and Logit-logistic) were generated and their parameters were selected so that the median/mean of probability distribution was equal to the pooled estimate of specificity/sensitivity, and the dispersion becomes consistent with 95% confidence intervals. The pooled results obtained for cancer and normal populations were used to determine the distribution parameters in differential misclassification bias analysis and the results for the total population were used to determine the distribution parameters in non-differential misclassification bias analysis. Table 1 presents the probability distribution parameters for Triangular, Beta and Logit-logistic distributions. It should be noted that the correlation of sensitivity and specificity was set to be 0.8, 0.5 and 0.25 in both case and control groups in differential misclassification bias analysis.

**Table 1:** The bias parameters for Triangular, Beta and Logit-logistic distributions in control and case groups

	Bias parameters (95% CI)		Group	Triangular distribution: min; max; mode	Beta distribution: alpha; beta	Logit-logistic distribution: location; scale
**Type of misclassification**	**Differential**	Sensitivity 65% (41, 89)	Case group	0.41; 0.89; 0.65	9.21; 4.96	0.65; 0.0675
		Specificity 93% (80, 100)		0.80; 1; 0.93	46.74; 3.51	0.93; 0.0196
		Sensitivity 54% (42, 65)	Control group	0.42; 0.65; 0.54	42.05; 35.82	0.54; 0.0309
		Specificity 90% (85, 100)		0.85; 1; 0.90	30.22; 3.36	0.90; 0.0281
	**Non-differential**	Sensitivity 60% (49, 77)	Both groups	0.49; 0.77; 0.60	18.54; 12.36	0.60; 0.0478
		Specificity 92% (77, 100)		0.77; 1; 0.92	39.87; 3.47	0.92; 0.0224

- Step 4: A sensitivity/specificity matrix was generated to estimate the expected number of exposed and unexposed cases according to Formula 1.

1 $$\begin{bmatrix}\text{Sen}&1-\text{Spe}\\1-\text{Sen}&\text{Spe}\end{bmatrix}\begin{bmatrix}\text{A}\\\text{B}\end{bmatrix}=\begin{bmatrix}\text{A}^\text{*}\\\text{B}^\text{*}\end{bmatrix}$$

where Sen and Spe refer to sensitivity and specificity, A is the expected number of exposed cases, B is the expected number of unexposed cases, A* is the observed number of exposed cases, and B* is the observed number of unexposed cases. Random values were selected for sensitivity and specificity from the probability distributions discussed in step 3 and plugged in Formula 1. Then, values A and B were obtained using Formula 1 based on Formulas 2 and 3: (see Supplement 2 for more explanations)

2 $$\mathrm{A}= [(\frac{\mathrm{Spe}}{\mathrm{Sen}+\mathrm{Spe}-1 } {\mathrm{)\times\ A}}^{*}]+[( \frac{\mathrm{Spe}-1}{\mathrm{Sen}+\mathrm{Spe}-1 } {\mathrm{)\times B}}^{*}]$$

3 $$\mathrm{B}= [(\frac{\mathrm{Sen}-1}{\mathrm{Sen}+\mathrm{Spe}-1 } {\mathrm{)\times A}}^{*}]+ [(\frac{\mathrm{Sen}}{\mathrm{Sen}+\mathrm{Spe}-1 } {\mathrm{)\times B}}^{*}]$$

- Step 5: Formulas 4 and 5 were used to calculate positive predictive value (PPV) and negative predictive value (NPV):

4 $$\mathrm{PPV}= \frac{\mathrm{(Sen \times A)}}{\mathrm{[(Sen \times A)]}+\left[(1-\mathrm{Spe}\right) \mathrm{\times B]}}$$

5 $$\mathrm{NPV}= \frac{\mathrm{(Spe \times B)}}{\mathrm{[(Spe \times B)]}+\left[(1-\mathrm{Sen}\right) \mathrm{\times A]}}$$

If there were out-of-range values for PPV and NPV (< 0 or > 1), the iteration process was discarded and steps 4 and 5 were repeated.

- Step 6: The status of observed exposure in dataset and PPV/NPV were used to generate a new variable termed “expected exposure” in cases. The distribution of this variable was Bernoulli with the probability parameters equal to PPV for exposed and NPV for unexposed cases. Therefore, a uniform random variable U_i_ ranging from 0 to 1 was generated. For an exposed case, the value of expected exposure was considered 1 (exposed) if U_i_<PPV and 0 (unexposed) otherwise. By contrast, for an unexposed case, the value of true exposure was considered 0 (unexposed) if U_i_<NPV and 1 (exposed) otherwise. Steps 4–6 were repeated for estimation of true exposure in controls.
- Step 7: The same conventional logistic regression model mentioned above was applied again using the expected exposure (alcohol consumption), generated through steps 1–6, instead of observed exposure, and adjusted OR with 95% confidence interval was reported.
- Step 8: The adjusted OR obtained in Step 7 resulted from one round of analysis. The steps 4–7 were repeated applying probabilistic bias analysis and the Monte-Carlo technique to obtain a simulation interval. This procedure corrects the misclassification bias in self-reported alcohol consumption. Then, the 50th percentile of the OR distribution was used as the point estimate and the 2.5th and 97.5th percentiles as the Monte-Carlo sensitivity analysis (MCSA) interval [29].

This point estimate with MCSA interval was only adjusted for misclassification bias and confounders. To address random error, the bootstrap sampling was performed prior to step 4 so that confounding and misclassification adjustment in steps 4–8 were applied to each bootstrap samples. The 95% MCSA intervals incorporating bias and random error were calculated using the 2.5th and 97.5th percentiles over all bootstrap-Monte-Carlo samples. It should be mentioned that there were 500 bootstrap samples, and Monte-Carlo was repeated 1000 times in each bootstrap sample yielding 500,000 adjusted ORs.

### Population Attributable Fraction (PAF)

The Miettinen Formula [61] was used for calculatiing PAF for alcohol consumption using Formula 6:

6 $$\mathrm{PAF}= \frac{{\mathrm{p}}_{\mathrm{e} }(\mathrm{RR}-1)}{\mathrm{RR}}$$

where ${p}_{e }$ is the prevalence of exposure in the case group and RR is the adjusted risk ratio. The proportion of alcohol consumers in the case group after misclassification bias correction in step 6 was used as ${p}_{e }$ estimate, and adjusted OR obtained in step 7 was considered as RR estimate based on the rarity assumption [62–64]. It is noteworthy that to calculate point estimate and MCSA interval for PAF, Monte-Carlo sampling and bootstrap technique were used.

## Result

This study was conducted in 1000 healthy controls and 932 cases. The mean SD age of participants was 42.16 (9.49) years old in the control group and 50.40 (9.70) in the case group. The characteristics of the case and control groups have been presented in Table 2.

**Table 2:** Characteristics of cases and controls

Variables	No. (31)	
	Control	Case
**Marital status**		
Married	792 (79.2)	744 (79.8)
Single	133 (13.3)	61 (6.5)
Divorced	34 (3.4)	47 (5.0)
Widow	41 (4.1)	80 (8.6)
**Insurance**		
No	107 (10.7)	32 (3.4)
Yes	893 (89.3)	900 (96.6)
**Education**		
Illiterate	48 (4.8)	55 (5.9)
Primary	108 (10.8)	163 (17.5)
Secondary	158 (15.8)	148 (15.9)
High school	342 (34.2)	316 (33.9)
Bachelor	284 (28.4)	200 (21.5)
More than bachelor	60 (6.0)	50 (5.4)
**Job**		
Housekeeper	723 (72.3)	746 (80.0)
Government employed	159 (15.9)	96 (10.3)
Self employed	108 (10.8)	48 (5.2)
Retired	10 (1.0)	42 (4.5)
**SES**		
Very low	11 (1.1)	25 (2.7)
Low	68 (6.8)	187 (20.1)
Middle	273 (27.3)	491 (52.7)
High	638 (63.8)	198 (21.2)
Very high	10 (1.0)	31 (3.3)
**Alcohol**		
No	971 (97.1)	892 (95.7)
Yes	29 (2.9)	40 (4.3)
**Physical activity**		
No	891 (89.1)	833 (89.4)
Yes	109 (10.9)	99 (10.6)
**Smoking**		
No	970 (97.0)	890 (95.5)
Yes	30 (3.0)	42 (4.5)
**Age**^**a**^	42.16 (9.49)	50.40 (9.70)

^a^mean and SD

**Fig. 3:**
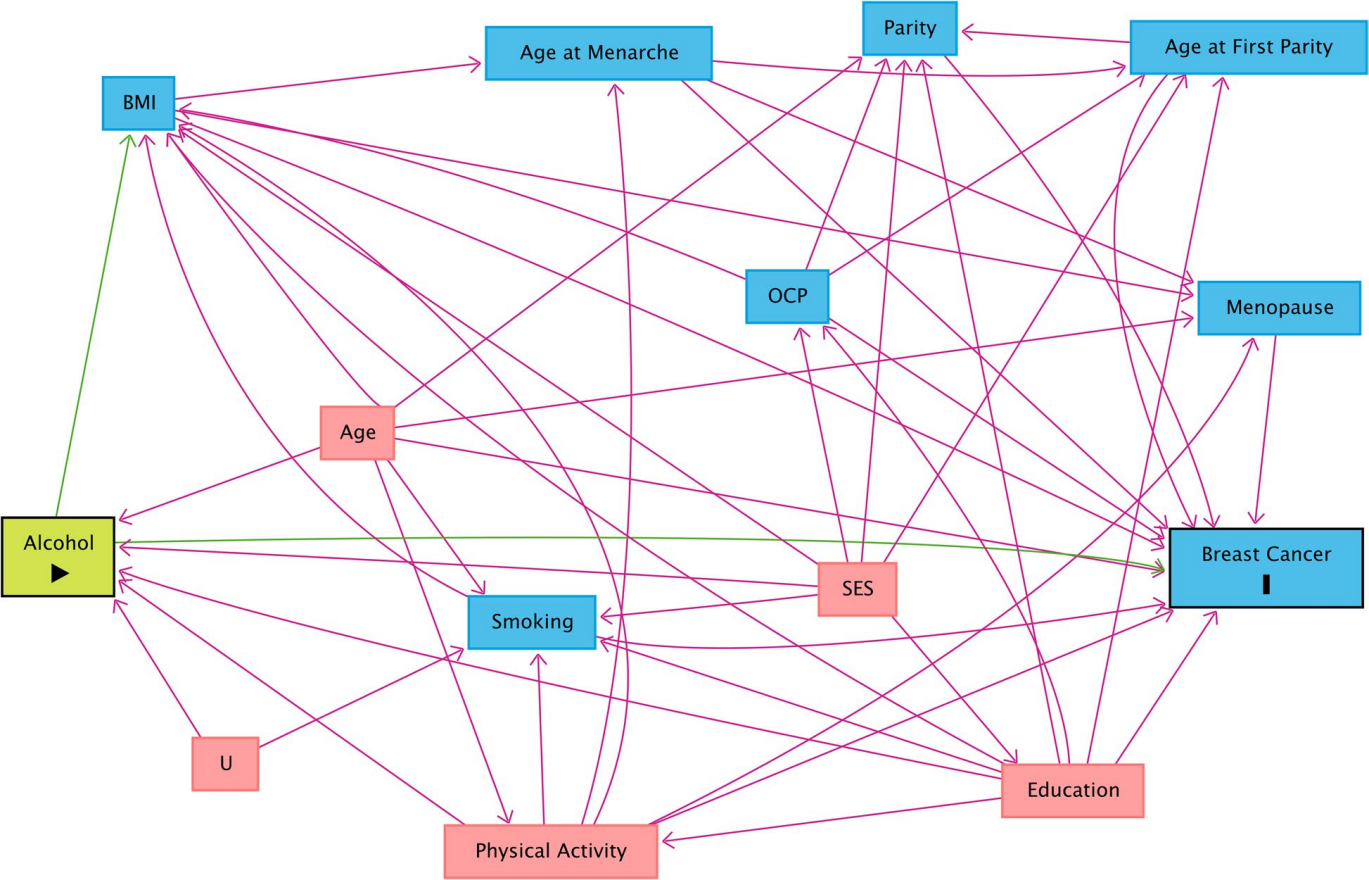
Causal directed acyclic graph (cDAG) for the effect of alcohol consumption on breast cancer

The causal diagram for the effect of alcohol consumption on breast cancer has been depicted in Fig. 3. According to this Figure, the minimally sufficient adjustment set included age, smoking, education level, physical activity, and socioeconomic status (SES).

### Conventional and bias analyses

**Fig. 4:**
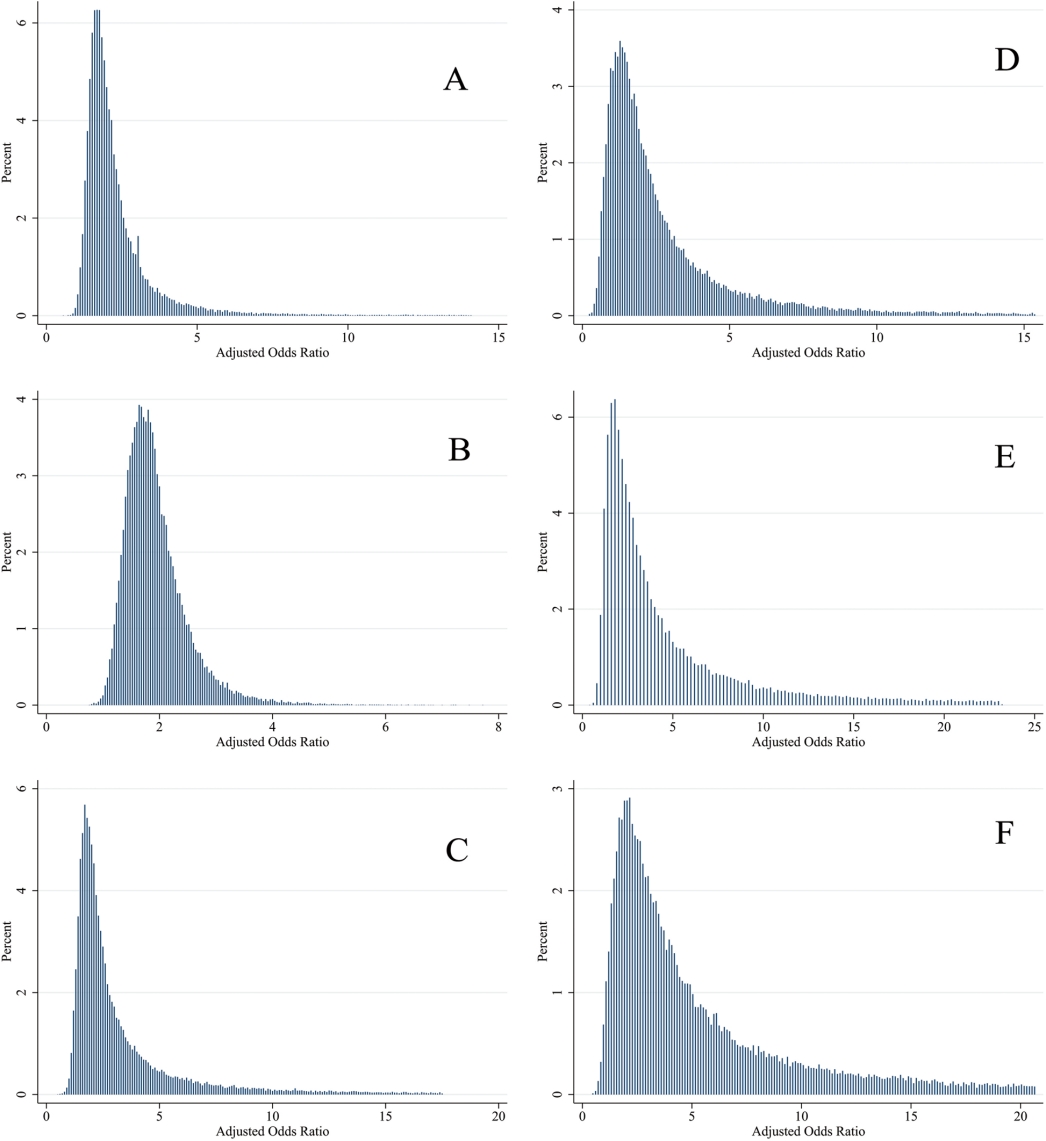
Distribution of ORs adjusted for measurement bias and confounding, assuming non-differential (**A**, **B** and **C**) and differential (**D**, **E** and **F**) misclassification errors. The distribution of bias parameter was assumed to be Triangular (**A** and **D**), Beta (**B** and **E**) and Logit-logistic (**C** and **F**)

Table 3 presents the results of the conventional and bias analyses for the effect of alcohol consumption on breast cancer. Based on the conventional logistic regression analysis, the OR between alcohol consumption and breast cancer was 1.05 (95% CI: 0.57, 1.91) implying no evidence against the independence of breast cancer from alcohol consumption. According to the results of bias analysis, considering non-differential misclassification, the adjusted estimate of OR was 1.96 (MCSA interval: 1.20, 6.01) using Triangular distribution, 1.82 (MCSA interval: 1.20, 3.38) using Beta distribution, and 2.29 (MCSA interval: 1.23, 11.84) using Logit-logistic distribution for the bias parameter, indicating that alcohol consumption was a risk factor for breast cancer. On the contrary, considering differential misclassification with correlation 0.8, the adjusted OR estimates were 1.93 (MCSA interval: 0.67, 10.07), 2.99 (MCSA interval: 1.44, 17.74) and 3.65 (MCSA interval: 1.16, 17.42) using the Triangular, Beta and Logit-logistic distributions, respectively. The distribution of adjusted ORs using different bias parameters has been displayed in Fig. 4.

**Table 3:** Adjusted odds ratio with 95% confidence interval or MCSA interval using conventional and probabilistic bias analyses. All estimates were obtained by adjusting for age, smoking, education level, physical activity and socioeconomic status

Conventional analysis	Bias parameter distribution	Bias analysis (95% MCSA)			
		Non-differential	Differential (*r* = 0.8)	Differential (*r* = 0.5)	Differential (*r* = 0.25)
1.05 (95% CI: 0.57, 1.91)	Triangular	1.96 (1.20, 6.01)	1.93 (0.67, 10.07)	2.03 (0.97, 12.10)	2.30 (1.28, 12.23)
	Beta	1.82 (1.20, 3.83)	2.99 (1.11, 17.74)	5.14 (3.53, 22.09)	5.56 (3.88, 24.00)
	Logit-logistic	2.29 (1.23, 11.84)	3.65 (1.16, 17.42)	5.20 (4.13, 18.00)	5.67 (4.64, 18.30)

### Population attributable fraction

Table 4 shows PAF estimates with 95% confidence intervals using conventional and bias analyses. PAF estimate for alcohol consumption was 0.20% (95% CI: -3.24, 2.50) in conventional analysis. Considering Triangular, Beta and Logit-logistic distributions for the bias parameter in non-differential bias analysis, the PAF estimates for alcohol consumption were 1.76% (MCSA interval: 0.31, 5.27), 1.51% (MCSA interval: 0.27, 4.19) and 2.57% (MCSA interval: 0.37, 9.32), respectively; in differential bias analysis with correlation 0.8, they were 1.54% (MCSA interval: -0.91, 5.92), 2.85% (MCSA interval: 0.21, 6.81) and 3.32% (MCSA interval: 0.41, 6.85). Other values for differential scenario were shown in Table 4.

**Table 4:** The estimates of population attributable fraction with 95% confidence intervals or MSCA intervals using conventional and bias analyses

Conventional analysis	Bias parameter distribution	Bias analysis (95% MCSA)			
		**Non-differential**	**Differential (** ***r*** ** = 0.8)**	**Differential (** ***r*** ** = 0.5)**	**Differential (** ***r*** ** = 0.25)**
0.20% (95% CI: -3.24, 2.50)	Triangular	1.76% (0.31, 5.27)	1.54% (-0.91, 5.92)	1.53% (-0.03, 5.92)	1.51% (0.23, 5.74)
	Beta	1.51% (0.27, 4.19)	2.85% (0.21, 6.81)	3.25% (0.20, 7.03)	3.56% (1.09, 7.21)
	Logit-logistic	2.57% (0.37, 9.32)	3.32% (0.41, 6.85)	2.57% (0.14, 6.34)	2.58% (0.15, 6.35)

## Discussion

In this study, we assess the effect of alcohol consumption on breast cancer after controlling three error sources including misclassification bias, confounders, and random error. The PBAM is a type of Monte-Carlo sensitivity analysis that is very similar to Bayesian methods [33, 65–67] and its results are affected by prior distributions [29, 33]. Therefore, the PBAM results depend on the distribution of sensitivity and specificity of the misclassified variable under question [68]. Using the same prior distributions for sensitivity and specificity parameters, the results of PBAM and Bayesian methods should be very similar [33]. Although different sources were used to determine the distribution of sensitivity and specificity, such as expert opinion and study validation, [26, 69] the medical literature seems to be one of the best sources [70]. The use of medical literature allows investigators to incorporate subjective data in their study while merging different sources can neutralize the effects of these judgments [29]. Different sources produce different results; therefore, in this study, to obtain more robust estimates of bias parameters, inverse variance weighting was used to merge these sources.

Based on the results of the conventional analysis in the present study, there was no evidence against independence of self-reported alcohol consumption and breast cancer. This finding was consistent with many previous reports [11–16]. However, it should be noted that case-control studies are prone to misclassification bias due to recall and underreporting [23, 71].

Cohort studies are much less prone to differential measurement error because exposure ascertainment occurs before the onset of the outcome (although differential measurement error can still occur due to dependence of exposure measurement on for some risk factors such as age) and prospective data collection should also reduce measurement error due to poor recall of past exposures [72]. However, similar to case–control studies of alcohol consumption and breast cancer, the results of cohort studies were inconsistent (the results have not been shown but available upon request).

Because of the inconsistent results and some limitations in primary studies, to evaluate the association between alcohol consumption and breast cancer, studies with higher levels of evidence, like meta-analyses, should be relied upon. Ziembicki et al. conducted a meta-analysis through merging 11 studies and found that alcohol consumption had a direct association with percent breast density. However, the effects of unmeasured confounders like smoking and measurement bias in alcohol consumption have not been controlled in the majority of meta-analyses [24]. Bagnardi et al. merged 49 studies and reported that alcohol consumption increased the risk of breast cancer; however, the authors discussed that they could not control the role of alcohol consumption underreporting and other confounders [17, 18]. Choi et al. merged 34 studies and found a positive association between alcohol consumption and breast cancer although this association was very weak (RR = 1.04; 95% CI: 1.01, 1.07), which was due to underreporting according to authors [25]. Another meta-analysis study found a positive association between alcohol consumption and breast cancer; nonetheless, they reported that misclassification and lack of adjustment for confounders were inevitable in primary studies [7].

The results of this study showed that alcohol consumption had a strong effect on breast cancer after adjusting misclassification bias and controlling confounders such as smoking. The range of adjusted OR estimates was 1.82 to 2.29 when controlling for non-differential misclassification and 1.93 to 5.67 when controlling for differential misclassification, suggesting that the effect the alcohol consumption was markedly underestimated if misclassification bias was not properly corrected.

Biologically, it seems that alcohol consumption increases epithelial cell proliferation resulting in dense tissue development in the breast through increased endogenous estrogen production [73], increased aromatase activity [74] and the components of the growth hormone-insulin-like growth factor [75] axis [76], resulting in increased risk of breast cancer [24].

A limited number of studies have used the PBAM for misclassification correction; hence, an extensive search failed to find similar results for comparison. However, this method has been applied in other studies with different context [4, 77–81]. De Silva et al. [79] reported a stronger association between maternal transfusion risk and inter-pregnancy interval after adjusting for severe maternal morbidity misclassification. One study [78] reported that the association between self-reported pre-pregnancy BMI and pregnancy outcomes was overestimated without considering misclassification. Pakzad et al. showed a strong association between smoking and breast cancer after smoking misclassification bias correction [4]. Nonetheless, Momoli et al. [80] and Bodnar et al. [77] found no marked change in the observed relationship after applying PBAM versus conventional methods.

This study estimated the PAF for alcohol consumption and breast cancer. It is clear that alcohol consumption is one of the most important risk factors of cancers. Daily consumption of up to 20 gr of alcohol (≤ 1.5 drinks) is responsible for 26–35% of alcohol-attributable cancer deaths [82]. Since PAF is a function of risk ratio (odds ratio for rare outcomes) and prevalence [83], its estimated prevalence may not show the actual prevalence because of alcohol consumption underreporting/recall bias. According to the recommendations of other studies [84], PAF calculation was done with misclassification correction. Based on the results, PAF ranged from 1.51 to 2.57% in non-differential bias analysis and from 1.54 to 3.56% in differential bias analysis. It means that if alcohol consumption had been eliminated, the risk of breast cancer would have been reduced by 1.5–3.6%. Van Gemert et al. found a PAF of 6.6% for alcohol consumption and breast cancer in the Netherlands [84]. Furthermore, Neutel et al. [85] conducted a study in Canadian women and estimated a PAF range of 2.7–2.6% for alcohol consumption and breast cancer during 1994–2006. PAF estimates for alcohol consumption were 2.8% and 6.4% in the Australian and UK woman in studies by Wilson et al. [86] and Parkin et al. [87], respectively. In a met-analysis by Key et al. [23], PAF estimates were 0.9–2.4% in the USA and 3.2–8.8% in the UK. There was a difference between the PAF estimates of the present study and the above studies, which could be secondary to differences in the prevalence of alcohol consumption in women.

The role of non-differential and differential misclassification was considered in this study. Differential exposure misclassification is more common in traditional case-control studies since the exposure data collection is done after disease diagnosis [26]. Considering a wide range of scenarios, in differential exposure misclassification, the correlation coefficient assumed to be 0.8, 0.5 and 0.25. The result showed that when correlation value increased, the result of differential misclassification will approach to that of non-differential misclassification.

Simple bias analysis can be performed by applying bias correction in each confounder stratum along with summarization. Nonetheless, this method takes a lot of time and does not consider the distribution of the bias parameters. Therefore, it may produce sparse data problems [69, 88, 89]. Other methods like empirical and Bayesian methods are more challenging in terms of calculations while bias correction can be done probabilistically in PBAM, considering distribution of bias parameters to impute the true exposure [29, 68]. This method is simpler and can be applied to estimate the association adjusted for multiple covariates using logistic regression, proportional hazards regression, and other popular modeling techniques [29]. In addition, Monte-Carlo simulations will make it possible to consider all misclassification sources resulting in more robust bias-adjusted estimates [68].

However, it should be noted that although Monte Carlo sensitivity analysis moved point estimates away from the null, the uncertainty interval were widened. In other words, taking into account the uncertainty due to measurement bias in the Monte Carlo approach led to a wider interval as expected.

A systematic search for the values of the bias parameter, using different distributions for the bias parameter, and assuming differential and non-differential misclassification error scenarios were some of the strong points of this study. In this study, a minimally sufficient adjustment set was detected using causal diagram [90] and their confounding bias was corrected using multiple logistic regression. To avoid over-adjustment bias, we did not adjust for the mediators on the pathway between alcohol consumption and breast cancer such as menopause or age at menopause. Finally, we carefully adjusted for the difference in age between cases and controls using LOWESS and fractional polynomials.

However, this study also suffered from some limitations. First there was some misclassification in using ever/never alcohol consumption instead of “number of drinks (bottle/can) of alcohol” which may reduce statistical power, induce a biased impression of dose-response, and change non-differential error to differential [91]. Also there was a considerable heterogeneity among included studies for the calculation of the bias parameters so the random-effects model was used. Moreover, the specificity in the case group was larger than in the control group, and only two studies for cancer patients were meta-analyzed to derive the bias parameters for the case group which is subject to small-sample bias. Also the studies in the meta-analysis were non-local and there is not a reliable study in Iran to determine how odds ratio will be changing by considering the local validations. In other words, it is difficult to perform a very meaningful adjustment for misclassification in the studied setting, and therefore validation studies specific to Iran seem warranted. Another limitation of the present study was inability to control for unmeasured confounding (e.g., diet) and misclassification in self-reporting confounders like smoking. We should note that presence of measurement error in a confounder like smoking will lead to residual confounding although our study objective was correcting alcohol consumption misclassification but not unmeasured confounding. We appreciate the misclassification error in smoking and alcohol is likely correlated which may increase the residual confounding [91]. However, the prevalence of smoking in women living in Tehran was 2.9% [92] and in Iranian woman was 3.6% [93] and so smoking probably cannot be a strong confounder (prevalence of smoking in our control group was 3%). We also calculated the E-value [94] i.e., the minimum strength of association, on the risk ratio (odds ratio for rare outcomes) scale, that an unmeasured confounder would need to have with both the exposure and outcome, conditional on the measured confounders, to fully explain away a specific exposure –outcome association. The results for different bias analysis scenarios have been presented in Table 5. The Table shows that smoking needs to have a large association (OR = 10.82 in one differential scenario) with both alcohol and breast cancer to fully explain the observed association between alcohol and breast cancer. It should be noted that the calculation of E-values assumes no adjustment was made for smoking although we did adjust for the self-reported smoking in the analysis.

**Table 5:** E-Values for alcohol assuming no adjustment was made for this variable

Bias parameter distribution	Bias analysis (95% MCSA )			
	Non-differential	Differential (*r* = 0.8)	Differential (*r* = 0.5)	Differential (*r* = 0.25)
**Triangular**	3.30 (1.68, 11.49)	3.27 (1.00, 19.62)	3.48 (1.00, 23.69)	4.03 (1.88, 23.95)
**Beta**	3.04 (1.69, 7.12)	5.43 (1.46, 34.93)	9.75 (6.52, 43.67)	10.61 (7.22, 47.49)
**Logit-logistic**	4.01 (1.76, 23.16)	6.76 (1.59, 34.33)	9.87 (7.73, 35.49)	10.82 (8.75, 36.09)

## Conclusion

Our conventional analysis showed no strong evidence of association between alcohol consumption and breast cancer although it is a well-known risk factor for several cancers. It seems that conventional analysis was unable to produce an unbiased estimate of association for sensitive exposures that are markedly prone to measurement error. According to PBAM, alcohol consumption was a strong risk factor for breast cancer with an OR of 1.82 to 5.67 in different scenarios. This study also found that 1.51–3.56% of breast cancers were attributed to alcohol consumption. Therefore, the breast cancer incidence can be reduced, although slightly in our population due to low prevalence of alcohol, through alcohol cessation programs. However, future confirmatory studies can provide more evidence for proper assessment of the effects of variables prone to misclassification bias and potentially encourage researchers to use PBAM methodology in the future.

## Supplementary Information

**Additional file 1: Supplement 1.** Characteristics of included studies for calculating the bias parameters.

**Additional file 2. **Mathematical logic for obtaining the expected values from the sensitivity-specificity matrix.

## Data Availability

The all datasets and codes used during the current study are available from the corresponding author on reasonable request.

## Abbreviations

BM: Bayesian Method
PBAM: Probabilistic Bias Analysis Method
cDAG: casual Directed Acyclic Graph
LOWESS: LOcally WEighted Scatterplot Smoother
CIs: Confidence Intervals
PPV: Positive Predictive Value
NPV: Negative Predictive Value
MCSA: Monte-Carlo Sensitivity Analysis
PAF: Population Attributable Fraction
SES: Socio-Economic Status
