# Supplementary material for: Effect of alcohol consumption on breast cancer: probabilistic bias analysis for adjustment of exposure misclassification bias and confounders
Source: BMC Med Res Methodol. 2023 Jul 4;23:157. doi: 10.1186/s12874-023-01978-6 (PMC10318777; doi:10.1186/s12874-023-01978-6)
Supplement: Supplementary file 1 — Additional file 1: Supplement 1. Characteristics of included studies for calculating the bias parameters. [file 12874_2023_1978_MOESM1_ESM.docx]

Supplement 1: characteristic of included strides for calculates the bias parameter

| study | Sample | Outcome | Case | Normal | Instrument | Sensitivity% (95% CI) | Specificity (95% CI) |
| --- | --- | --- | --- | --- | --- | --- | --- |
| Osório et al. | All cancer | Alcohol abuse | 40 | 360 | Self-report against FASD (>=4) | 58.10 (41 to 73) | 86 (82 to 90) |
|  |  | Alcohol dependence | 21 | 379 |  | 48.02 (26 to 71) | 83 (79 to 87) |
| Bonevski et al. | Cancer | Any alcohol per week | 12 | 103 | Urine marker | 86.03 (77 to 95) | 95 (91 to 98) |
|  |  |  | 8 | 208 |  |  |  |
| Fleming et al. | Alcohol vs. normal population | Any alcohol per week | 56 | 52 | Blood | 70.58 (58.29 to 81.02) | 94.73 (88.14 to 8.26) |
| Williams et al | Alcohol vs. normal population | Any alcohol per week | 17 | 321 | Urinalysis | 11.76 (1.45 to 36.44) | 98.13 (95.97 to 9.31) |
| Baggio et al. | Normal population | Alcohol use | 78 | 155 | Blood | 60.26 (48.53 to 71.16) | 87.10 (8.77 to 91.93) |
| Van de Luitgaarden et al. | Normal population | Alcohol use | 3214 | 2997 | Urinalysis | 47.00 (45 to 49) | 95 (95 to 96) |
| Oppolzer et al. | Normal population | Alcohol use | 782 | 188 | Urinalysis | 79.98 (64 to 90) | 90 (65 to 98) |
| Karns-Wright et al. | Normal population | Alcohol use | 244 | 478 | Computer monitoring | 62.02 (51 to 72) | 74 (60 to 85) |
| May et al. | Normal population | Alcohol use | 125 | 68 | Blood biomarker | 40.10 (31.3 to 49.1) | 75 (63 to 84) |
