# Supplementary material for: Effect of alcohol consumption on breast cancer: probabilistic bias analysis for adjustment of exposure misclassification bias and confounders
Source: BMC Med Res Methodol. 2023 Jul 4;23:157. doi: 10.1186/s12874-023-01978-6 (PMC10318777; doi:10.1186/s12874-023-01978-6)
Supplement: Supplementary file 2 — Additional file 2. Mathematical logic for obtaining the expected values from the sensitivity-specificity matrix. [file 12874_2023_1978_MOESM2_ESM.docx]

**Supplement 2.** Mathematical logic for obtaining the expected values from the sensitivity-specificity matrix

Consider the following matrix:

$$\left[ \begin{matrix} Sen & 1-Spe \\ 1-Sen & Spe \end{matrix} \right]* \left[ \begin{matrix} A \\ B \end{matrix} \right]= \left[ \begin{matrix} A^{*} \\ B^{*} \end{matrix} \right]$$

This matrix shows that the observed values in the case group (i.e. $A^{*}$ and $B^{*}$) is equal to the result of multiplying the sensitivity/specificity matrix ($\left[ \begin{matrix} Sen & 1-Spe \\ 1-Sen & Spe \end{matrix} \right]$) by the value of expected exposure in the case group ($\left[ \begin{matrix} A \\ B \end{matrix} \right]$). The above matrix was resorted for expected exposure calculation, which resulted in the following matrix:

$$\left[ \begin{matrix} A \\ B \end{matrix} \right]=\left[ \begin{matrix} A^{*} \\ B^{*} \end{matrix} \right]* \left[ \begin{matrix} Sen & 1-Spe \\ 1-Sen & Spe \end{matrix} \right]^{-1}$$

According to matrix rules, reverse of matrix is equal to

$$G=\left[ \begin{matrix} a & b \\ c & d \end{matrix} \right] => G^{-1}= \frac{1}{ad-bc} \left[ \begin{matrix} d & -b \\ -c & a \end{matrix} \right]$$

Therefore, reverse of sensitivity/specificity matrix would be

$$\left[ \begin{matrix} Sen & 1-Spe \\ 1-Sen & Spe \end{matrix} \right]^{-1}= \frac{1}{\left( Sen*Spe \right)- \left[ \left( 1-Sen \right)* \left( 1-Spe \right) \right]}*\left[ \begin{matrix} Spe & -\left( 1-Spe \right) \\ -\left( 1-Sen \right) & Sen \end{matrix} \right]= \frac{1}{Sen+Spe-1}*\left[ \begin{matrix} Spe & Spe-1 \\ Sen-1 & Sen \end{matrix} \right]= \left[ \begin{matrix} \frac{Spe}{Sen+Spe-1} & \frac{Spe-1}{Sen+Spe-1} \\ \frac{Sen-1}{Sen+Spe-1} & \frac{Sen}{Sen+Spe-1} \end{matrix} \right]$$

So by plugging above result in formula we can write that:

$$\left[ \begin{matrix} A \\ B \end{matrix} \right]=\left[ \begin{matrix} \frac{Spe}{Sen+Spe-1} & \frac{Spe-1}{Sen+Spe-1} \\ \frac{Sen-1}{Sen+Spe-1} & \frac{Sen}{Sen+Spe-1} \end{matrix} \right]* \left[ \begin{matrix} A^{*} \\ B^{*} \end{matrix} \right]$$

Accordingly, A and B would be calculated using the following formulas:

$$A= \frac{Spe}{Sen+Spe-1} A^{*}+ \frac{Spe-1}{Sen+Spe-1} B^{*}$$

$$B= \frac{Sen-1}{Sen+Spe-1} A^{*}+ \frac{Sen}{Sen+Spe-1} B^{*}$$

The above calculations can be done for the control group too.
